# Activation of the MET receptor attenuates doxorubicin‐induced cardiotoxicity in vivo and in vitro

**DOI:** 10.1111/bph.15039

**Published:** 2020-05-29

**Authors:** Simona Gallo, Martina Spilinga, Raffaella Albano, Giuseppe Ferrauto, Enza Di Gregorio, Elena Casanova, Davide Balmativola, Alessandro Bonzano, Carla Boccaccio, Anna Sapino, Paolo Maria Comoglio, Tiziana Crepaldi

**Affiliations:** ^1^ Candiolo Cancer Institute FPO‐IRCCS Candiolo (TO) Italy; ^2^ Department of Oncology University of Turin Turin Italy; ^3^ Department of Molecular Biotechnology and Health Sciences University of Turin Turin Italy; ^4^ Department of Medical Sciences University of Turin Turin Italy

## Abstract

**Background and Purpose:**

Doxorubicin anti‐cancer therapy is associated with cardiotoxicity, resulting from DNA damage response (DDR). Hepatocyte growth factor (HGF) protects cardiomyocytes from injury, but its effective use is compromised by low biodistribution. In this study, we have investigated whether the activation of the HGF receptor—encoded by the *Met* gene—by an agonist monoclonal antibody (mAb) could protect against doxorubicin‐induced cardiotoxicity.

**Experimental Approach:**

The mAb (5 mg·kg^−1^) was injected in vivo into C57BL/6J mice, before doxorubicin (three doses of 7 mg·kg^−1^). Cardiac functions were evaluated through MRI after treatment termination. Heart histological staining and mRNA levels of genes associated with heart failure (*Acta1* and *Nppa*), inflammation (*IL‐6*), and fibrosis (*Ctgf*, *Col1a2*, *Timp1*, and *Mmp9*) were assessed. MAb (100 nM) was administered in vitro to H9c2 cardiomyoblasts before addition of doxorubicin (25 μM). DDR and apoptosis markers were evaluated by quantitative western blotting, flow cytometry, and immunofluorescence. Stattic was used for pharmacological inactivation of STAT3.

**Key Results:**

In vivo, administration of the mAb alleviated doxorubicin‐induced cardiac dysfunction and fibrosis. In vitro, mAb mimicked the response to HGF by (a) inhibiting histone H2AX phosphorylation at S139, (b) quenching the expression of the DNA repair enzyme PARP1, and (c) reducing the proteolytic activation of caspase 3. The MET‐driven cardioprotection involved, at least in vitro, the phosphorylation of STAT3.

**Conclusion and Implications:**

The MET agonist mAb provides a new tool for cardioprotection against anthracycline cardiotoxicity.

**Abbreviations**
DDR: DNA damage response
HGF: hepatocyte growth factor
JNJ: JNJ‐38877606
mAb: monoclonal antibody agonist
γH2AX: S139 phosphorylated histone H2AX

**What is already known**

- Anti‐tumour treatments with the anthracycline doxorubicin engender unwanted side effects such as cardiotoxicity.
- Doxorubicin‐induced cardiotoxicity involves activation of DNA damage response and apoptosis of cardiomyocytes.

**What this study adds**

- A monoclonal antibody with agonist activity at MET receptors prevents doxorubicin‐induced cardiotoxicity in vivo.
- DNA integrity protecting and survival‐promoting activity of HGF–MET is mediated by STAT3 in vitro.

**What is the clinical significance**

- Agonist monoclonal antibody produces MET receptor‐mediated protection in a preclinical model of cardiotoxicity.
- This monoclonal antibody could be developed as a treatment for anthracycline‐induced cardiotoxicity.

## 1 INTRODUCTION

The anthracycline doxorubicin is a potent, widely used anti‐neoplastic drug effective in several forms of cancer, such as sarcoma, leukaemia, lymphoma, breast, ovarian, and lung cancer (Curigliano et al., 2016). However, its clinical use is limited by serious side effects, with cardiac toxicity being the most prominent (Gianni et al., 2008; Tacar, Sriamornsak, & Dass, 2013). Cardiotoxicity is dose‐dependent, is characterized by left ventricular dysfunction, may develop several years after treatment, and may end in heart failure. Earlier studies have shown that even the so‐called safe dose of doxorubicin (<300 mg·m^−2^) increases patient risk (Swain, Whaley, & Ewer, 2003).

The anti‐cancer activity of doxorubicin depends on its inhibition of α topoisomerase II, involved in DNA replication and cell proliferation (Pommier, Sun, Huang, & Nitiss, 2016). However, the cardiotoxicity involves different molecular mechanisms, including interaction with iron, generation of ROS, alteration of calcium homeostasis, and inhibition of β topoisomerase II, which is highly expressed in myocardium (Stěrba et al., 2013). β topoisomerase II, doxorubicin and DNA form a ternary cleavage complex that induces double‐strand breaks, activates the DNA damage response (DDR), and triggers cell death (Lyu et al., 2007; Zhang et al., 2012).

Recently, we and others showed that the MET tyrosine kinase, the receptor for the hepatocyte growth factor (HGF), plays a key role in cardiac protection against hypoxic injury (Gallo et al., 2014). Silencing the *Met* gene in cardiomyocytes enhances the age‐induced accumulation of ROS (Arechederra et al., 2013). In addition, HGF drives migration and proliferation of cardiac stem cells (Urbanek et al., 2005). These results suggest that HGF may exert beneficial cardioprotective functions in other contexts of stress or injury. Unfortunately, the use of HGF in therapy is severely limited by its poor bioavailability, as a consequence of its binding to heparan and dermatan sulfates of the extracellular matrix (Lyon et al., 1998). Here, we demonstrate that activation of MET, by means of an agonist monoclonal antibody (mAb), alleviates doxorubicin‐induced cardiotoxicity.

## 2 METHODS

### 2.1 Animals, treatment and sample collection

All animal care and experimental procedures were approved by the Ethical Commission of the Candiolo Cancer Institute, FPO‐IRCCS, and by the Italian Ministry of Health (867/2017‐PR). The preclinical studies involving animals were performed in accordance with the National Centre for the Replacement, Refinement and Reduction of Animals in Research. Animal studies are reported in compliance with the ARRIVE guidelines (Kilkenny et al., 2010) and with the recommendations made by the *British Journal of Pharmacology.*

We chose the *Mus musculus* model, as it presents genetic, metabolic, and functional affinities with the human species but low neurological development. We excluded female mice, as they are better protected from cardiovascular complications, relative to male animals, to obtain homogeneous animal sample groups. A limitation of our study is that results cannot be extrapolated to female mice and this aspect will be investigated in the future.

Adult male C57BL/6J mice (RRID:IMSR_JAX:000664; 4 months of age, average weight: 30 g) were purchased from Charles River Laboratories (Wilmington, MA, USA). The animals were housed in the specific pathogen free animal facility at Candiolo Cancer Institute in groups of no more than five mice and monitored daily. Environmental enrichments were routinely used in the cages to improve the animal welfare. The animal rooms were maintained under a constant 12‐hr light/dark cycle at 23°C and relative humidity of 50 ± 10%. Mice were allowed ad libitum access to standard pellets and water. All animals were acclimatized for 2 weeks before the experiments.

Mice were randomized into three groups: placebo‐treated, doxorubicin‐treated (Doxo), and a group treated with doxorubicin and the agonist mAb (Doxo + mAb) (Figure S1). Mice were treated with a total of three i.p. injections of placebo (sterile saline solution; placebo) or doxorubicin (7 mg·kg^−1^; Doxo and Doxo + mAb) every 7 days. The cumulative dose of doxorubicin was 21 mg·kg^−1^ (or ~180 mg·m^−2^) at the end of treatment. In addition, the Doxo + mAb group received a further i.p. injection of mAb (5 mg·kg^−1^) the day before each doxorubicin administration. Animals were weighed once a week. The mice gradually lost body weight on doxorubicin treatment (less than 20%) without signs of overt suffering. At 35 days after the initiation of doxorubicin treatment, the mice were anaesthetized (isoflurane) and killed by cervical dislocation.

For western blot analysis of mouse hearts (Figure 3c), a cohort of animals was untreated (placebo) or treated with a single i.p. injection of doxorubicin (15 mg·kg^−1^; Doxo and Doxo + mAb). Doxo + mAb mice received mAb (5 mg·kg^−1^) 24 hr before drug administration, and all mice were killed 48 hr after doxorubicin administration. For treatment of mice, the dose of doxorubicin was chosen on the basis of published results (Bartlett, Trivedi, & Pulinilkunnil, 2017). To mimic the long‐term doxorubicin cardiotoxicity, animal models are usually treated with a cumulative drug dose ranging from 15 to 36 mg·kg^−1^ (Bartlett et al., 2017; Wang et al., 2014; Yi et al., 2006). For these reasons, we chose a cumulative dose of 21 mg·kg^−1^. For signalling pathway analysis, animals were treated once with 15 mg·kg^−1^ doxorubicin and killed 48 hr after treatment.

Organs were immediately rinsed in ice‐cold PBS solution (Sigma‐Aldrich, Saint Louis, MO, USA), grossly dried, weighed, and immersed in RNAlater (Sigma‐Aldrich) overnight at 4°C, and then stored at −80°C, to preserve total proteins. Hindlimbs were excised and digested overnight with Proteinase K (Sigma‐Aldrich). Tibias were scanned and measured using ImageJ (RRID:SCR_003070). The mean tibia lengths were used for normalization of organ weights.

### 2.2 MRI

MRI of mice from the three experimental groups (placebo, *n* = 8; Doxo, *n* = 10; and Doxo + mAb, *n* = 12) was performed 28 days after the initiation of Doxo treatment. The trained operator and data analysis were blinded to the experimental groups. MRI analysis was carried out by using a Bruker Avance 300 spectrometer (B_0_ = 7 T) equipped with a Micro 2.5 microimaging probe. Mice were anaesthetized by intramuscular injection of a mixture of 20 mg·kg^−1^ tiletamine/zolazepam (Zoletil 100; Virbac, Milan, Italy) plus 5 mg·kg^−1^ xylazine (Rompun; Bayer, Milan, Italy). In order to gain insights into the doxorubicin‐induced cachexia, morphological T_2w_ magnetic resonance images of body trunk were acquired by using a typical “rapid acquisition with refocused echoes MRI sequence” with the following parameters: repetition time = 5,000 ms; echo time = 5.5 ms; rapid acquisition with refocused echoes factor = 16; field of view = 35 mm × 35 mm; slice thickness = 1 mm; matrix size = 256 × 256; in‐plane spatial resolution = 0.137 × 0.137 mm^2^ per pixel; number of parallel slices = 9; and number of average = 8. The regions of interest were manually drawn in each slice, and the volumes of spino‐trapezius muscles were measured. Cardiac function was assessed by acquiring cardio‐magnetic resonance images with the same MRI scanner. For reliable electrocardiographic synchronization and high‐resolution heart imaging, a dedicated small animal electrocardiographic device (1025‐MR, SA Instruments Inc., Stony Brook, NY, USA) was used. Heart rate was monitored by ECG. At the beginning of examination, several T_2w_ scout images were acquired in the transverse plane and the long‐axis plane of the left ventricle to determine the orientation of the short axis. The localization of the central short‐axis slice was planned half‐way between the apex and the base. The whole heart was covered by acquiring five to seven short‐axis parallel slices (slice thickness = 1 mm; number of slices has been chosen in order to cover the entire heart). CINE MRI was carried out by using an electrocardiographic‐triggered FLASH CINE gradient‐spoiled gradient echo sequence with the following parameters: flip angle = 15°; repetition time = 8 ms; echo time = 2.5 ms; field of view = 35 mm × 35 mm; matrix size = 192 × 192; in‐plane spatial resolution = 0.182 × 0.182 mm^2^ per pixel; slice thickness = 1 mm; number of average = 6; and heart cycle sampled with 20 images. After the acquisition, morphological parameters of interest for cardiac function were measured in diastole and systole (Bakermans et al., 2015; Grabmaier et al., 2014). In particular, left end diastolic volume (EDV) and left end systolic volume (ESV) were manually measured. Then left stroke volume (SV), cardiac output (CO), and ejection fraction (EF%) were calculated by applying the following formula: SV = EDV − ESV; EF% = ((SV/EDV) × 100); and CO = HR × SV.

### 2.3 Masson's trichrome staining

PBS‐rinsed hearts were fixed in freshly made formalin (Sigma‐Aldrich) overnight at 4°C and then processed with four cuts parallel and transversal to the tip, the midline of the ventricles, the base, and the atria. After processing, the tissues were embedded in paraffin (Leica Microsystems, Wetzlar, Germany); 5‐μm‐thick serial sections were deparaffinized, rehydrated, and stained with haematoxylin and eosin (Bio‐Optica, Milan, Italy). Heart horizontal sections at midlevel were also stained with Masson's trichrome staining kit (Bio‐Optica), following the manufacturer's protocol. Images were taken with Leica ICC50 microscope (Leica Microsystems) at 40× magnification. LAS AF Leica software (Leica Microsystems) was used for acquisition. Five images for sample (a total of 25 images for group of analysis) were used to quantify the Masson blue staining through ImageJ software.

### 2.4 mRNA quantitative analyses

Total RNA was extracted from mouse hearts (*n* = 5 per group) with Maxwell RCS miRNA Tissue Kit, according to the manufacturer's protocol (Promega Corporation, Madison, WI, USA). The extracted RNA was quantified with NanoDrop, and the reverse transcription was performed with iScript Reverse Transcription Supermix (Biorad, CA, USA). Quantitative PCR assay was performed on an ABI 7500 Fast Real‐Time PCR System using TaqMan Fast Universal PCR master mixture and TaqMan Gene Expression Assay Probes (Applied Biosystems, Waltham, MA, USA). For probe references, see Table S2. PCR reactions were performed in triplicate for each sample and normalized to Polr2a gene expression.

### 2.5 Western blot analyses

Mouse heart ventricles (*n* = 5 per each experimental group) and H9c2 cells (*n* = 5 per each experimental group) were lysed in ice‐cold RIPA added with protease inhibitor cocktail (Sigma‐Aldrich). Lysates were subsequently sonicated and centrifuged at 12,000 *g* at +4°C (40 min for heart tissue and 20 min for cells). The protein concentration was evaluated with the BCA Protein Assay Kit (Thermo Fisher Scientific, Waltham, MA, USA). Proteins and prestained protein ladder (10–180 kDa, PageRuler™, Thermo Fisher Scientific) were separated by electrophoresis using precast 4–12% SDS‐PAGE gels (Invitrogen, Carlsbad, CA, USA) and transferred to Hybond‐P pvdf membrane (Bio‐Rad, Hercules, CA, USA). After incubation in blocking solution (10% BSA, Sigma‐Aldrich) at room temperature, membranes were incubated overnight at +4°C with the primary antibodies (see Table S3). Primary antibodies were diluted in BSA 5% TBS Tween and reused at most three times. ERK2 for in vivo analysis and α tubulin for in vitro analysis were used as loading control. Bands obtained from the same blot as the target protein of interest were exploited for normalization. Membranes were washed and then incubated with specific HRP‐conjugated secondary antibodies (Jackson Laboratory, Bar Harbor, ME, USA) for 1 hr at room temperature. Secondary antibodies were diluted in TBS Tween and used once only. The proteins were revealed by enhanced chemiluminescence of the ECL Prime detection kit and quantified with the Image Lab software (Bio‐Rad). Quantitation of band density was conducted, blindly, on analysis within the linear range. The immuno‐related procedures used comply with the recommendations made by the *British Journal of Pharmacology* (Alexander et al., 2018).

### 2.6 Cell culture and treatments

H9c2 cells were cultured in DMEM (Sigma‐Aldrich) supplemented with 10% FBS (Sigma‐Aldrich), 1% penicillin (Sigma‐Aldrich), 1% streptomycin (Sigma‐Aldrich), and 1% l‐glutamine (Sigma‐Aldrich) and were incubated under 5% CO_2_ at 37°C. Cells were passed regularly and subcultured to ~80/90% of confluence; 24 hr before the onset of the treatment, cells were cultured in low serum medium (0.5% FBS). For in vitro treatments, doxorubicin was used at 25 μM for 1 hr, a dose leading to full‐blown caspase 3 activation and induction of γH2AX and PARP1 (Figure S5a). HGF (0.5 nM), mAb (100 nM), and the inhibitors, JNJ‐38877605 (JNJ, 500 nM) and Stattic (10 μM), were administered 3 hr before doxorubicin treatment. Then the cells were maintained in fresh low serum medium for a further 24 hr (Figure S5b).

### 2.7 Immunofluorescence analysis

Cells were plated in fibronectin (3 μg·ml^−1^, Sigma‐Aldrich)‐coated 24‐well plates, fixed with ice‐cold 4% paraformaldehyde (Santa Cruz Biotechnology, Dallas, TX, USA) dissolved in PBS for 10 min, and washed with PBS. Fixed cells were permeabilized with 0.1% Triton X‐100 (Sigma‐Aldrich). Then the cells were saturated with 1% BSA (Sigma‐Aldrich) and incubated with the primary antibody (see Table S3) for 1 hr at room temperature. Secondary antibody incubation was performed with the Alexa Fluor 488‐conjugated goat anti‐rabbit antibody (Invitrogen) for 1 hr at room temperature. DNA was counterstained with DAPI and added at the end of secondary antibody incubation for 5 min at room temperature. Both primary and secondary antibodies were diluted in PBS 1% donkey serum and used once only. Cleaved caspase 3 images and quantitative images for γH2AX were taken through the Leica AF 6000LX workstation (Leica Microsystems). Representative images of γH2AX were taken by the Leica TCS SP2 AOBS confocal laser‐scanning microscope (Leica Microsystems). In both cases, images were processed with LAS AF software (Leica Microsystems). For the immunofluorescence quantification, the ImageJ software was used for counting the green positive cells and normalizing to the nuclei number. Five independent experiments were performed (45 fields per experimental condition). For γH2AX foci quantification, the green nuclei with high number of fluorescent spots/nucleus were considered positive and counted by manual scoring (200 nuclei being analysed per experiment).

### 2.8 Flow cytometric analysis

H9c2 cells were treated with FIX & PERM reagents (ADG Wien, Austria) and then stained for 30 min at room temperature, in the dark, with the anti‐γH2AX (Ser139) rabbit antibody (9718, Cell Signalling, Leiden, Netherlands, RRID:AB_2118009) and anti‐cleaved caspase‐3 rabbit antibody (559565, BD Pharmingen, San Diego, CA, USA, RRID:AB_397274). Then secondary antibody incubation was performed with the anti‐rabbit IgG (H + L) APC (4050‐11S, Southern Biotech, Birmingham, AL, USA, RRID:AB_2795959) for 30 min at room temperature, in the dark. Samples were analysed on a CyAn™ ADP LX nine‐colour analyser (Beckman Coulter, Brea, CA, USA).

### 2.9 Experimental design

**FIGURE 1:**
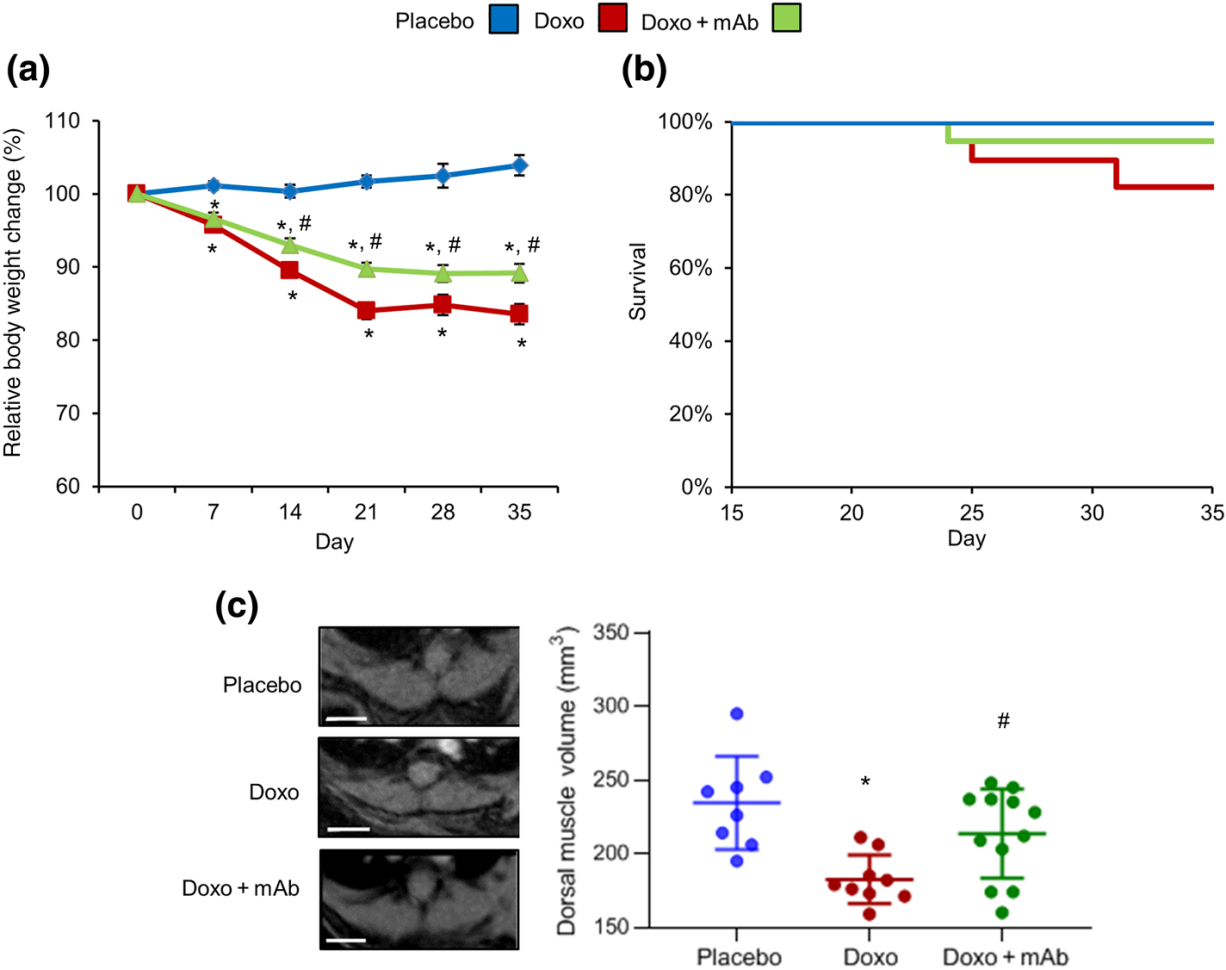
The agonist mAb reduces the cachectic effect mediated by doxorubicin treatment. Adult mice were treated with placebo (*n* = 8), doxorubicin (DOXO; 7 mg·kg^−1^, once per week up to 3 weeks; *n* = 10), or Doxo + mAb (5 mg·kg^−1^ for 24 hr before each doxorubicin treatment; *n* = 12). For treatment protocols, see Figure S1. (a) Body weight measurements and (b) survival rates. (c) Representative images (left panels) and volume quantification (right scatterplot) of spino‐trapezius muscles calculated by magnetic resonance analysis at Day 28 after onset of doxorubicin treatment. Bars: 3 mm. *P < .05, significantly different from placebo; ^#^ *P* < .05, significantly different from Doxo group

**FIGURE 2:**
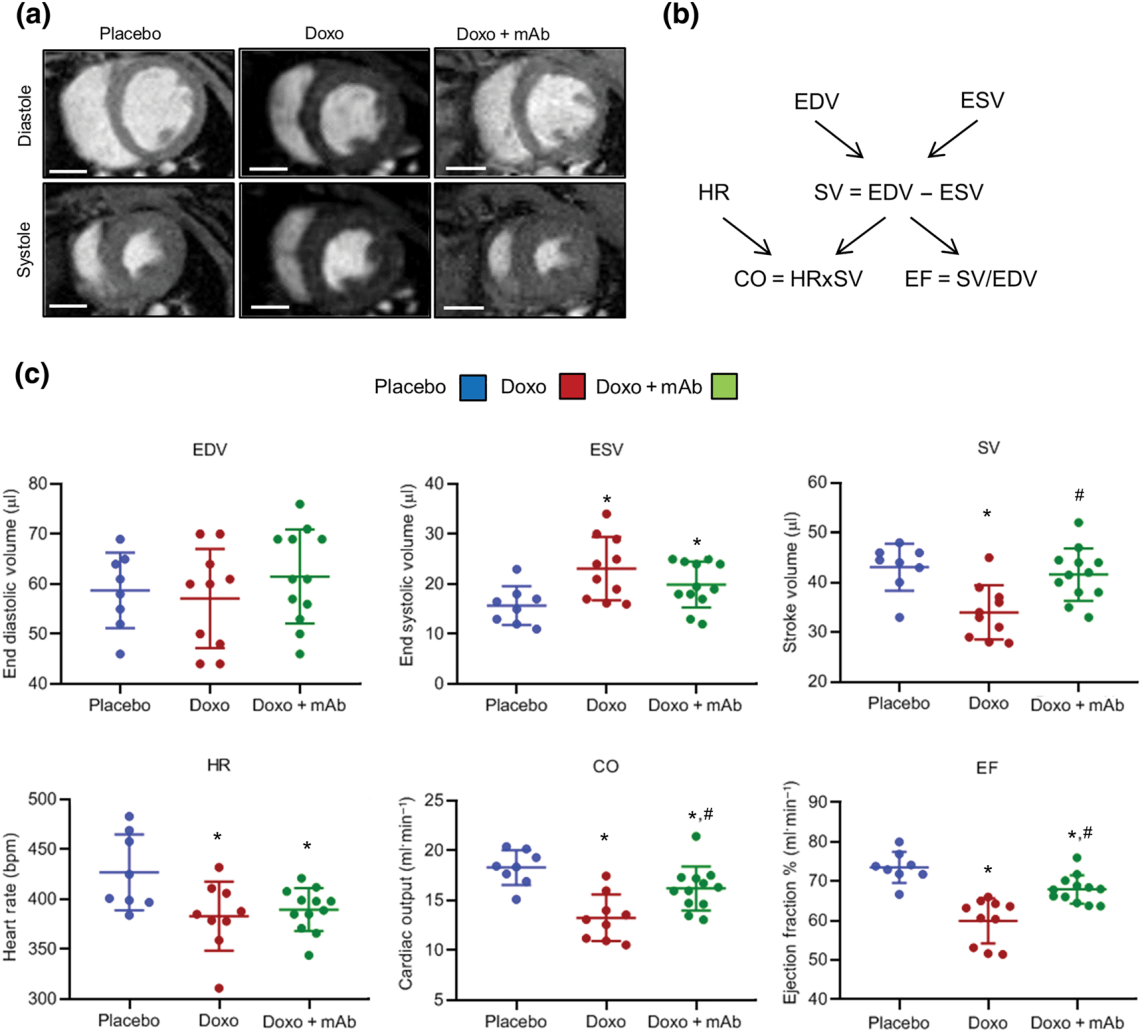
MAb attenuates the doxorubicin‐induced cardiac dysfunction. (a) Representative diastolic and systolic MRI of left ventricles from placebo, Doxo, and Doxo + mAb mice analysed at Day 28 after onset of doxorubicin treatment. For mice treatments, see Figure S1. Bars: 3 mm. (b) Schematic diagram explaining the existing correlation among cardiac functional parameters evaluated by magnetic resonance analysis of left ventricles and formulas utilized to calculate the values reported in (c). The parameters reported are end diastolic volume (EDV), end systolic volume (ESV), stroke volume (SV), heart rate (HR), cardiac output (CO), and ejection fraction (EF). (c) Representation of MRI parameters changes in the three experimental groups (placebo, *n* = 8; Doxo, *n* = 10; and Doxo + mAb, *n* = 12). *P < .05, significantly different from placebo; ^#^ *P* < .05, significantly different from Doxo group

The sample groups were formed randomly. Group sizes were designed to be equal (in the majority of the experiments, *n* = 5). An exception was for in vivo MRI analysis (Figures 1 and 2). As doxorubicin treatment could lead to animal death, we produced more animals for the Doxo groups (*n* = 13 vs. *n* = 8 for placebo). Three animals died in the Doxo group and one animal died in the Doxo + mAb group. This resulted in 10 animals for Doxo and 12 mice for Doxo + mAb available to evaluate by MRI analysis.

**FIGURE 3:**
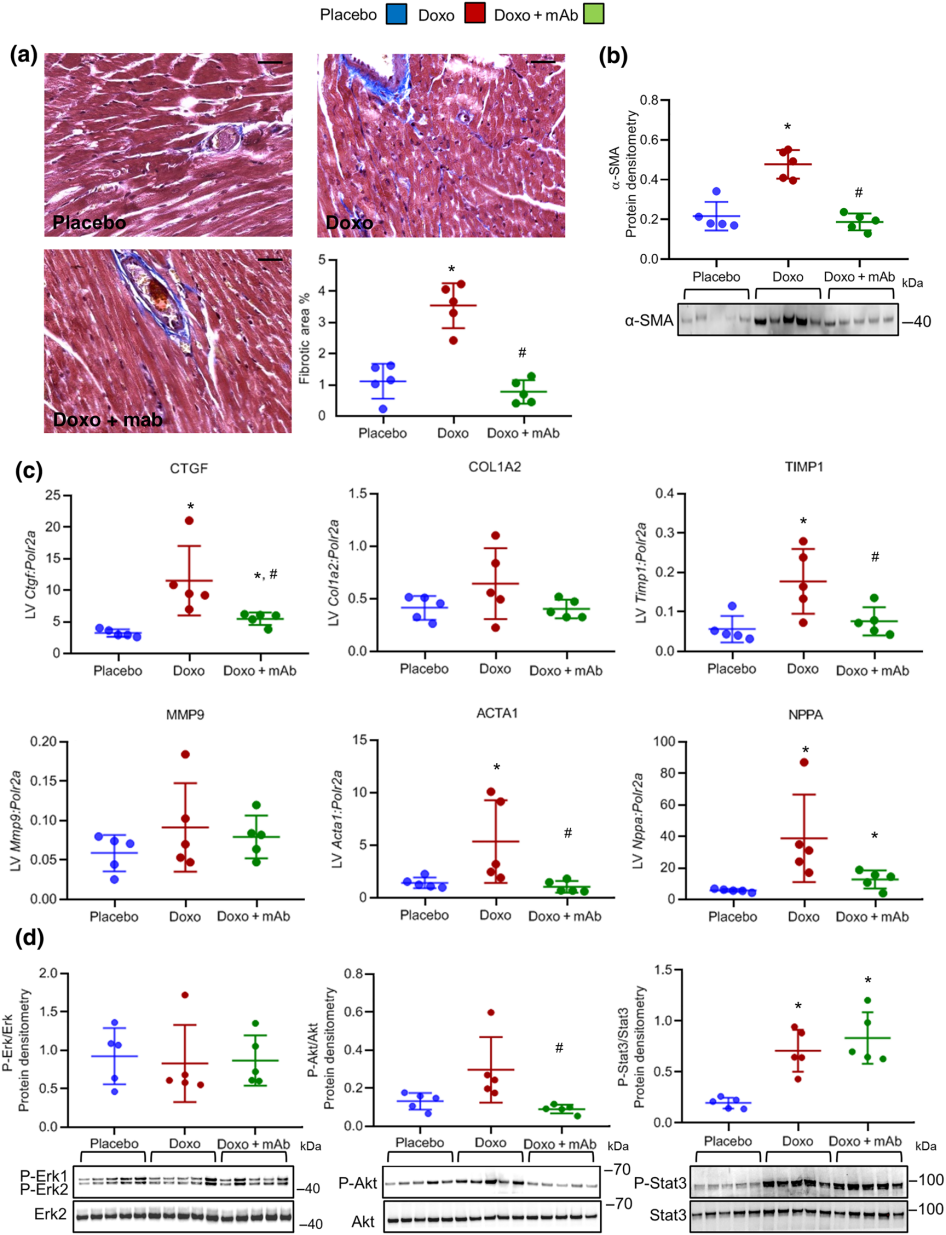
The agonist mAb exerts cardioprotective effects towards doxorubicin‐induced cardiotoxicity. (a) Representative images and quantification analysis of Masson's trichrome staining on hearts of placebo, Doxo, and Doxo + mAb mice at Day 35 after onset of doxorubicin treatment. Bars: 25 μm. (b) Western blot quantitative protein densitometry (above) and representative image (below) of α‐smooth muscle actin (α‐SMA) protein in heart tissues (*n* = 5 per group). (c) Gene expression analysis of connective tissue growth factor (Ctgf), collagen type I α 2 chain (Col1a2), TIMP metallopeptidase inhibitor 1 (Timp1), metallopeptidase 9 (Mmp9), α‐actin (Acta1), and atrial natriuretic peptide (Nppa) in the three cohorts of animals (*n* = 5 per group). Polr2a was used as reference gene for the expression data normalization. (a–c) For mice treatments, see Figure S1. (d) Western blot analysis was performed on heart lysates from mice of three treatment groups (placebo, Doxo, and Doxo + mAb; *n* = 5 per group). Doxorubicin (15 mg·kg^−1^) was injected i.p. as a single dose, and mAb (5 mg·kg^−1^) was administered 24 hr before doxorubicin. Mice were killed 48 hr after doxorubicin treatment. Quantitative protein densitometry (above) and representative images (below) of phosphorylated ERK1/2, Akt, and STAT3. ERK2 was used as loading control in all western blots. *P < .05, significantly different from placebo; ^#^ *P* < .05, significantly different from Doxo group

### 2.10 Data and statistical analysis

**FIGURE 4:**
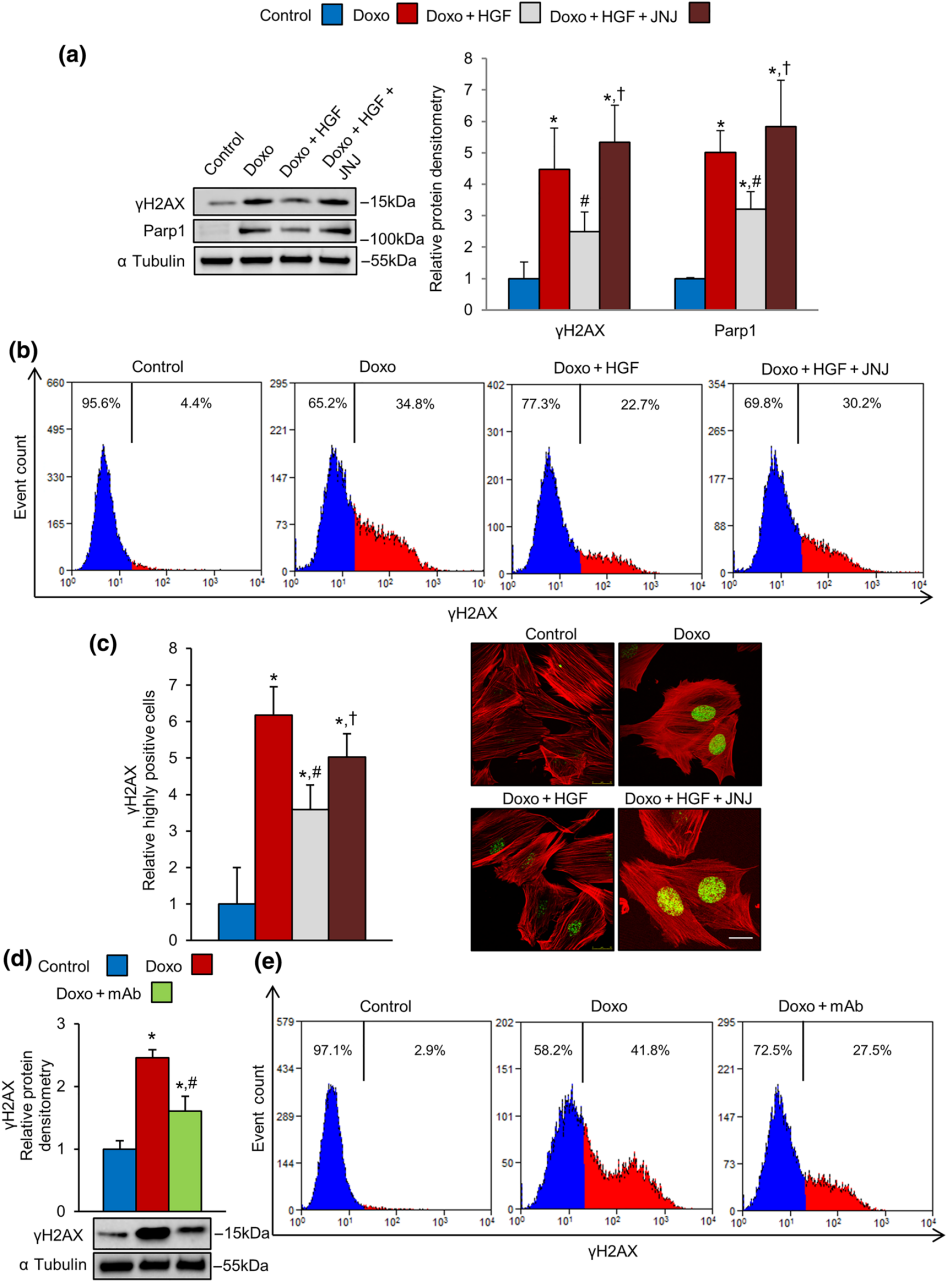
MET activation reduces doxorubicin‐induced DNA damage in H9c2 cardiomyoblasts. (a–c) H9c2 cells were untreated (control) or treated with doxorubicin (Doxo, 25 μM), Doxo + HGF (0.5 nM), and Doxo + HGF + JNJ (500 nM). For cell treatments, see Figure S5b. (a) Protein levels of γH2AX and PARP1 were measured by western blot (left) and densitometric (right) analysis. (b) γH2AX protein levels were measured by flow cytometry. (c) γH2AX foci were analysed and counted by immunofluorescence as described in Section 2. Representative images of F‐actin (red) and γH2AX (green) co‐staining show weakly (left panels) and highly (right panels) positive nuclei. Bar: 20 μm. (d, e) H9c2 cells were pretreated with 100‐nM mAb instead of the natural ligand, HGF. The level of γH2AX protein was analysed by (d) western blot (densitometry on the top and representative image on the bottom) and (e) flow cytometry; α tubulin was used as loading control in all western blots. All experimental data were obtained from five independent experiments. *P < .05, significantly different from control; ^#^ *P* < .05, significantly different from Doxo; †*P* < .05, significantly different from Doxo + HGF

**FIGURE 5:**
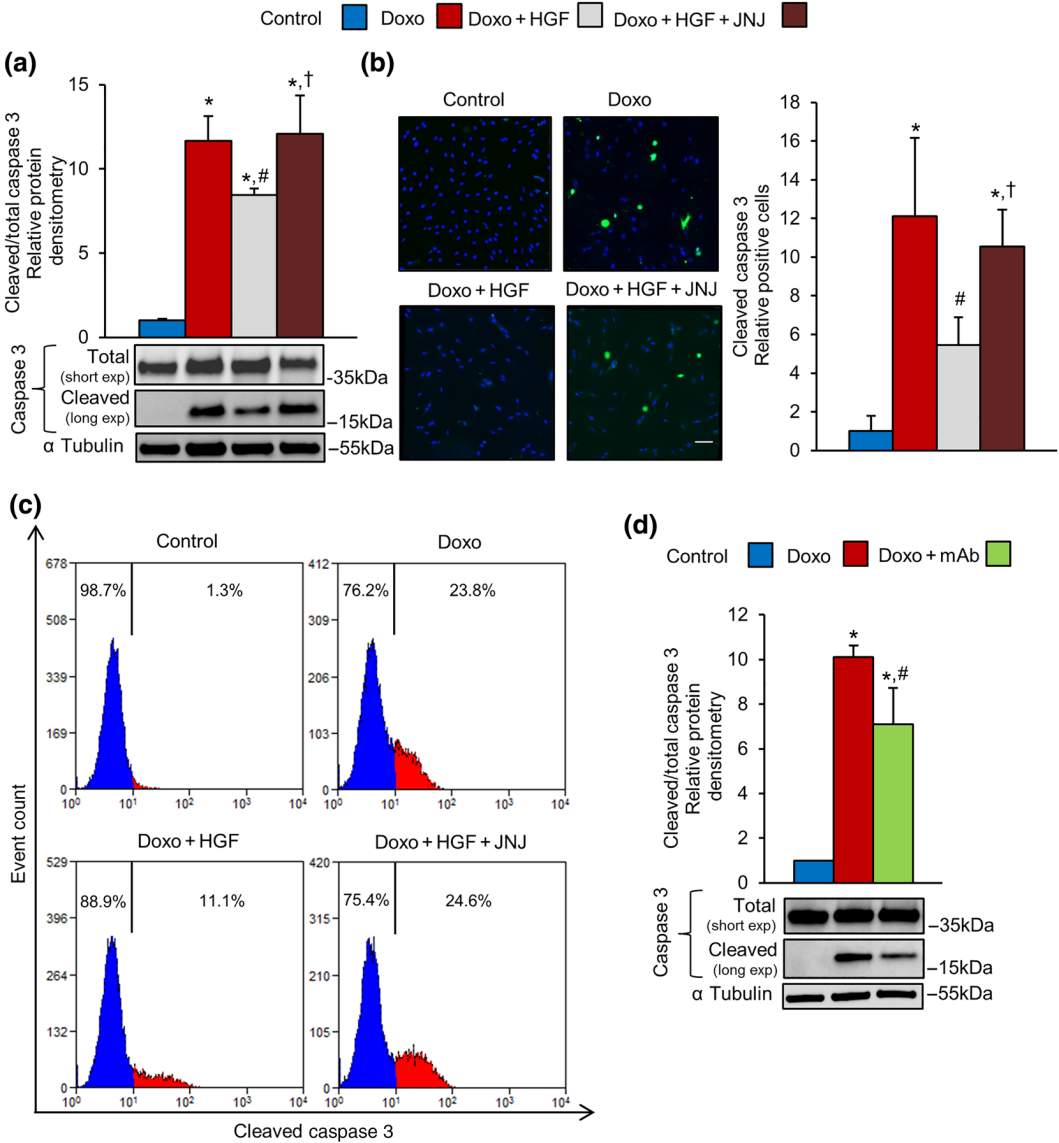
MET activation attenuates doxorubicin‐induced apoptosis in H9c2 cardiomyoblasts. (a–c) H9c2 cells were untreated (control) or treated with doxorubicin (Doxo, 25 μM), Doxo + HGF (0.5 nM), and Doxo + HGF + JNJ (500 nM). Cleaved caspase 3 apoptotic marker was quantified by (a) western blots, (b) immunofluorescence (cleaved caspase 3 green and DAPI blue; bar: 100 μm), and (c) flow cytometry. (d) H9c2 cells were pretreated with 100‐nM mAb instead of the natural ligand, HGF. The level of cleaved caspase 3 protein was determined by western blot (densitometry on the top and representative image on the bottom). Total and cleaved caspase 3 proteins (a, d) were evaluated using two different exposure times; α tubulin was used as loading control in all western blots. All experimental data were obtained from five independent experiments. *P < .05, significantly different from control; ^#^ *P* < .05, significantly different from Doxo; † *P* < .05, significantly different from Doxo + HGF

**FIGURE 6:**
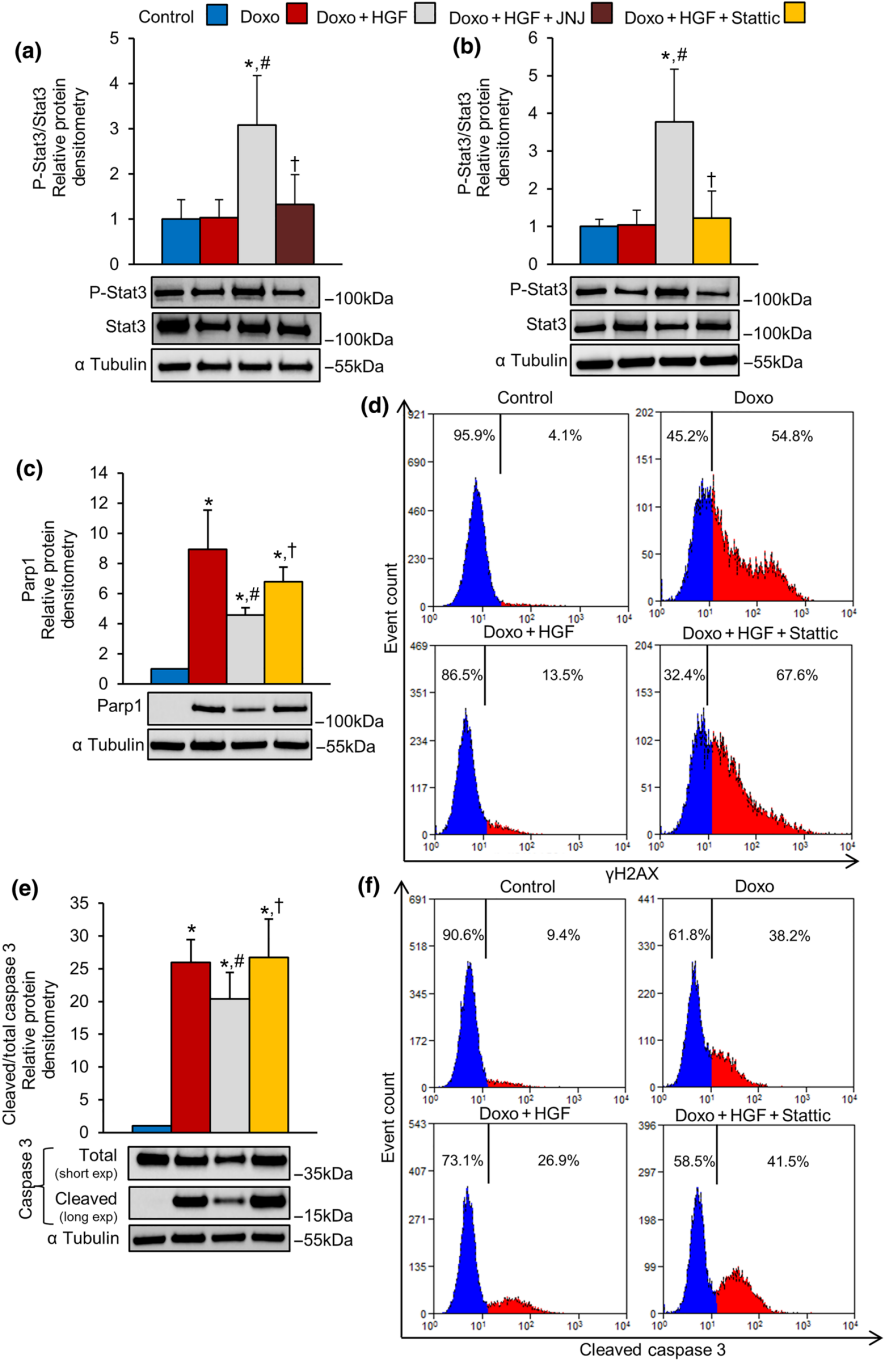
Activation of MET exerts cardioprotective effect towards doxorubicin via STAT3. (a) H9c2 cells were untreated (control) or treated with doxorubicin (DOXO, 25 μM), Doxo + HGF (0.5 nM), and Doxo + HGF + JNJ (500 nM). Protein levels of P‐STAT3 and STAT3 were analysed by western blot (densitometry above and representative image below). (b–f) H9c2 cells were untreated (control) or treated with Doxo (25 μM), Doxo + HGF (0.5 nM), and Doxo + HGF + Stattic (STAT3 inhibitor, 10 μM). Protein levels of (b) P‐STAT3 and total STAT3, (c) PARP1, and (e) total and cleaved caspase 3 were quantified by western blots (densitometric analysis (above) and representative immunoblot (below). Total and cleaved caspase 3 proteins were evaluated using two different exposure times; α tubulin was used as loading control. (d) γH2AX and (f) cleaved caspase 3 protein levels were measured by flow cytometry. All experimental data were obtained from five independent experiments. *P < .05, significantly different from control; ^#^ *P* < .05, significantly different from Doxo; †*P* < .05, significantly different from Doxo + HGF

All values are expressed as the mean ± SD of *n* independent experiments. The indicated group size refers to independent experiments and samples. Statistical analysis was performed blindly on these independent values and on groups with sample size at least of 5. For multiple comparisons, one‐way ANOVA was used, followed by the Tukey post hoc test. The post hoc test was performed only if the *F* was significant and there was no variance inhomogeneity. The threshold *P* value deemed to constitute statistical significance was <.05. Only data characterized by *P* value <.05 were denoted throughout the paper as results with statistical significance. In statistical analysis, all the samples were analysed, and the outliers were not excluded. The data analysis and scatterplots were made using the GraphPad Prism (RRID:SCR_002798) software. For in vitro analysis on H9c2 cells (Figures 4, 5, 6), we performed data transformation expressing the values as “fold mean control.” We applied this data protocol as the numbers of samples hampered the overall evaluation in a single experiment (i.e., electrophoresis gels or immunofluorescence staining). Thus, to compare different independent analyses, we quantified every evaluation as “fold mean control,” and then we combined the values and performed the statistical analysis. In figures, the “fold mean control” was indicated as “relative protein densitometry” and “relative positive cells.” The data and statistical analysis comply with the recommendations of the *British Journal of Pharmacology* on experimental design and analysis in pharmacology (Curtis et al., 2018).

### 2.11 Materials

The rat cardiomyoblast cell line H9c2 (RRID:CVCL_0286) was purchased from the American Type Culture Collection (Manassas, VA, USA). The DO24 mAb was produced as previously described (Prat, Crepaldi, Pennacchietti, Bussolino, & Comoglio, 1998). Doxorubicin hydrochloride (Pfizer, New York, NY, USA) was obtained from the Pharmacy of Candiolo Cancer Institute, FPO‐IRCCS. HGF was acquired from Tebu‐Bio (Le‐Perray‐en‐Yvelines, France). The MET tyrosine kinase inhibitor JNJ‐38877605 (JNJ) was kindly provided by Janssen Pharmaceutica (Beerse, Belgium). The STAT3 inhibitor Stattic was purchased from Sigma‐Aldrich.

### 2.12 Nomenclature of targets and ligands

Key protein targets and ligands in this article are hyperlinked to corresponding entries in http://www.guidetopharmacology.org, the common portal for data from the IUPHAR/BPS Guide to PHARMACOLOGY (Harding et al., 2018), and are permanently archived in the Concise Guide to PHARMACOLOGY 2019/20 (Alexander, Fabbro et al., 2019a, 2019b; Alexander, Kelly et al., 2019).

## 3 RESULTS

### 3.1 Activation of the MET receptor mitigates doxorubicin‐induced cachexia in vivo

As mentioned in Section 1, a serious side‐effect of doxorubicin, as well as other members of the anthracycline family, is induction of toxicity in muscles and parenchymal organs (Tacar et al., 2013). Adult male C57BL/6J mice were randomized into two groups and treated with a total of three doses of placebo or 7 mg·kg^−1^ doxorubicin every 7 days. The cumulative dose of doxorubicin reached 21 mg·kg^−1^ (or ~180 mg·m^−2^) at the end of treatment. This dose reproduces the clinical administration profile of doxorubicin as a chemotherapeutic drug in humans (Bartlett et al., 2017). At baseline, the cohorts of mice had similar body weights (placebo: 30.5 ± 3.1 g; Doxo: 32.3 ± 2.5 g). Following administration of doxorubicin, and without significant changes in survival rates, mice started losing body weight at Day 7 reaching a 20% total loss after 35 days (26.9 ± 2.1 vs. 32.3 ± 2.5 g; Figure 1a,b). To assess skeletal muscle wasting, the volumes of spino‐trapezius muscles were measured by acquiring T_2w_ magnetic resonance images. At Day 28 after initiation of doxorubicin treatment, muscle volume was significantly impaired (182.4 ± 5.5 vs 234.4 ± 11.2 mm3; Figure 1c). Doxorubicin caused significant weight loss (normalized to tibia length) of spleens, kidneys, and livers, but not in heart or lungs (Table S1). These observations are in keeping with clinical manifestations observed in patients treated with doxorubicin (Tacar et al., 2013).

According to the known trophic and cytoprotective effect of the HGF–MET axis, we tested a MET antibody (mAb) which is an agonist, i.e. it mimics the effect of HGF stimulation (Gallo et al., 2014). Mice received the mAb (5 mg·kg^−1^) 24 hr before each administration of doxorubicin. The Doxo + mAb group showed a 10% decrease of the initial body mass (28.1 ± 1.8 vs. 31.6 ± 1.8 g), which was significantly less than the cachectic effect of doxorubicin alone (Doxo group; Figure 1a). Notably, mAb treatment prevented the decline of dorsal muscle volume (213.5 ± 8.7 mm3; Figure 1c). In contrast, mAb did not prevent the doxorubicin‐mediated damage of parenchymal organs (Table S1). These results indicate that activation of the MET axis mediates a selective protective effect on skeletal muscles, alleviating doxorubicin‐mediated cachexia.

### 3.2 Activation of the MET receptor protects against the cardiotoxic effects of doxorubicin in vivo

In the clinic, the cumulative anthracycline dose often affects the function of the left ventricle (Davis & Witteles, 2013). To evaluate cardiac functions, magnetic resonance analysis was performed at Day 28 after initiation of the doxorubicin treatment (Figure 2). Representative cardiac MRI are shown in Figure 2a, and left ventricle functional parameters are reported in Figure 2b,c. Doxorubicin treatment increased ESV and decreased SV and CO as compared to controls, indicating impairment of the contraction force. These parameters were all restored to normal by the agonist antibody. EF was reduced after doxorubicin treatment, and again, mAb exerted a significant protective effect. Altogether, these results demonstrate that MET activation preserved cardiac contractility in the context of damage after chronic doxorubicin.

Histopathological analysis at Day 35 after initiation of doxorubicin treatment did not show evidence of necrosis or infiltrating immune cells in the hearts of doxorubicin‐treated animals (Figure S2a). However, a moderate, though not significant, increase of mRNA for the proinflammatory cytokine IL‐6 was detected in heart tissues given doxorubicin treatment (Figure S2c). Cardiomyocytes, stained by haematoxylin and eosin, were morphologically similar in all three groups (Figure S2b). Quantitative analysis of heart Masson's trichrome staining revealed a significant increase in perivascular and interstitial fibrosis in the Doxo group compared to the placebo‐ and Doxo + mAb‐treated mice (Figure 3a). In line with this observation, protein levels of α‐smooth muscle actin, another marker of fibrosis, were up‐regulated by doxorubicin and reduced by mAb treatment (Figure 3b). Moreover, gene expression analyses revealed that the mRNA levels of extracellular matrix proteins (connective tissue growth factor, collagen type I α 2 chain, metallopeptidase inhibitor (TIMP)1, and metallopeptidase 9) were up‐regulated in the heart of doxorubicin‐treated mice (Figure 3c). The same holds true for skeletal muscle α‐actin and atrial natriuretic peptide, which are early hallmarks of heart failure (Figure 3c). Importantly, mAb treatment prevented the elevation of these genes involved in fibrosis, cardiac remodelling, and contractile dysfunction (Figure 3c). Neither the Doxo group nor the Doxo + mAb group of mice showed any changes in the mRNA levels of MET receptor and HGF (Figure S3). To analyse the signalling pathway induced by MET activation, we produced an acute mouse model, using a single injection of doxorubicin. In fact, the molecular pathways induced by mAb treatment to counteract the damage induced by doxorubicin were undetectable after 3 weeks from the last treatment (long‐term cardiotoxicity mouse model, data not shown). In the acute model, animals were treated with a single i.p. injection of doxorubicin (15 mg·kg^−1^), and the Doxo + mAb mice received mAb (5 mg·kg^−1^) 24 hr before drug administration. Phosphorylation levels of the signal transducers in the pathway, downstream of MET, in heart tissues were examined 48 hr after doxorubicin treatment. Phosphorylation of ERK1/2 (ERK1 at T202 and Y204; ERK2 at T183 and Y185) was unaffected by doxorubicin and mAb treatments (Figure 3d). A slight activation of Akt (at S473), a major player in the PI3K/Akt pathway, was detected after doxorubicin treatment, relative to placebo and Doxo + mAb treatment (Figure 3d). A marked increase in phosphorylation of STAT3 (at Y705) was observed after doxorubicin treatment and was maintained in the Doxo + mAb group, compared with placebo (Figure 3d). Histochemical immunostaining of heart tissue showed that MET and, to a much lesser extent, STAT3 proteins were both expressed in cardiomyocytes (Figure S4), suggesting that cardiomyocytes are likely to be the target cells responding to MET‐mediated antibody protection.

### 3.3 Activation of the MET receptor protects cardiomyoblasts against doxorubicin‐induced DNA damage in vitro

To evaluate the direct effect of MET activation on cardiomyocytes, we used the H9c2 cardiomyoblasts in three experimental groups Doxo, Doxo + HGF, and Doxo + mAb. In cardiomyoblast cells, doxorubicin /β topoisomerase II/DNA complexes induce double‐strand breaks, which in turn activate a strong stress response known as the DDR (Huelsenbeck et al., 2011). Severe DNA damage fuels persistent DDR signalling and enrichment for factors orchestrating DNA repair. Phosphorylation of histone H2AX on S139 (γH2AX) is triggered by DDR, promoting the recruitment of PARP1 to repair DNA lesions (Luo & Kraus, 2012). We observed that 1‐hr exposure of H9c2 cells to doxorubicin (25 μM), followed by 24‐hr recovery, stimulated a strong DDR, as shown by increased levels of γH2AX and PARP1 proteins in western blots, compared with controls (Figure 4a). Cardiomyoblasts were pretreated for 4 hr with HGF, and doxorubicin was added in the last hour (Figure 4a). Activation of MET by HGF significantly attenuated the DDR response (Figure 4a). In line with the above observations, the levels of γH2AX protein, increased by doxorubicin alone, were reduced by HGF, as shown by flow cytometry measurements (Figure 4b). The induction of nuclear γH2AX signals and formation of γH2AX foci, characteristic hallmarks of DNA lesions (Rothkamm et al., 2015), were visualized and quantified by fluorescence microscopy imaging (Figure 4c). In line with western blotting and flow cytometry analysis, quantitative imaging of γH2AX foci showed that MET activation reduced doxorubicin‐induced DNA damage in cardiomyoblasts (Figure 4c). As a mirror experiment, pharmacological inactivation of MET by the specific inhibitor JNJ‐38877605 (JNJ) dampened the protection provided by MET against the genotoxic stress (Figure 4a–c). Superimposable results were obtained using the agonist mAb (100 nM) to activate the MET receptor (Figure 4d,e).

### 3.4 Activation of the MET receptor attenuates doxorubicin‐induced apoptosis in cardiomyoblasts in vitro

Sustained doxorubicin‐induced DNA damage triggers cell death by stimulation of the apoptotic pathway, involving the activation of the caspase cascade (Roos, Thomas, & Kaina, 2016). We confirmed that 1‐hr exposure to doxorubicin, followed by 24‐hr recovery, triggered a marked increase of cleaved caspase 3, a generally accepted marker of apoptosis (Figure 5a). Cardiomyoblasts were pretreated for 4 hr with HGF, and doxorubicin was added in the last hour (Figure 5a–c). HGF significantly reduced the level of cleaved caspase 3 protein (Figure 5a). In the mirror experiment, specific inactivation of MET by the JNJ inhibitor abolished protection from apoptosis (Figure 5a). Quantitative immunofluorescence imaging and flow cytometry measurements with two different anti‐active caspase 3 antibodies showed similar results (Figure 5b,c). The agonist mAb (100 nM), activating the MET receptor, generated a superimposable inhibition of the caspase 3 cleavage (Figure 5d).

### 3.5 STAT3 plays a key role in MET‐mediated cardioprotection in vitro

We tested the hypothesis that STAT3, a known signal transducer downstream of MET, (Boccaccio et al., 1998) plays a role in MET‐driven cardioprotection against doxorubicin toxicity. First, we analysed the phosphorylation level of STAT3 in the context of treatment with doxorubicin. H9c2 cardiomyoblasts were exposed to doxorubicin for 1 hr and allowed to recover for 24 hr. STAT3 was found basally phosphorylated in both control and doxorubicin‐treated cells (Figure 6a). HGF further enhanced and the specific JNJ inhibitor blunted the phosphorylation of STAT3(Figure 6a). Consistent with these findings, the MET agonist mAb (100 nM) stimulated STAT3 phosphorylation in doxorubicin‐treated H9c2 cardiomyoblasts (Figure S6). As the mAb has lower affinity for the MET receptor than the natural ligand, the extent of STAT3 phosphorylation by the mAb was less than that after HGF treatment. We then tested the STAT3‐specific inhibitor, Stattic, a small molecule that prevents STAT3 dimerization and nuclear translocation (Schust, Sperl, Hollis, Mayer, & Berg, 2006). Direct inhibition of STAT3 prevented the MET‐induced decrease of PARP1 and γH2AX (Figure 6b–d). Moreover, the STAT3 inhibitor fully blocked the MET‐induced cardioprotection, measured by quantitative western blot and flow cytometry with anti‐cleaved caspase 3 antibodies (Figure 6e,f). Altogether, these results provide evidence for STAT3 as a leading player in the anti‐genotoxic and pro‐survival pathways activated by the Met receptor.

## 4 DISCUSSION

Doxorubicin induces severe side effects in many organs, including cachexia, and may severely affect the quality of life in cancer patients. Here, we showed that activation of the MET receptor by an agonist mAb provides a useful strategy to protect against the toxicity of the drug in a preclinical model system. To mimic the anthracycline‐based chemotherapy protocol in patients, we set up an animal model with chronic administration of doxorubicin, reaching a cumulative drug dose (21 mg·kg^−1^) comparable to that used clinically. Administration of the agonist mAb attenuated weight loss following doxorubicin, suggesting a potential use in prevention of muscle wasting. MET signalling is, in fact, essential for development and regeneration of skeletal muscles (Prunotto et al., 2004; Webster & Fan, 2013)and we have reported that protection of muscle progenitor cells from apoptosis is achieved by MET activation (Cassano et al., 2008). How chemotherapy causes cachexia is not known: Doxorubicin may exert adverse effects on skeletal muscle directly and/or indirectly by inducing cardiac dysfunction, as heart failure exacerbated muscle wasting (Anker et al., 1997). In cancer patients, the agonist mAb may break the vicious circle established between the damaged heart and skeletal muscles.

The natural ligand for MET is HGF, a large protein produced as a biologically inactive precursor activated by specific convertases (Naldini et al., 1992). Moreover, it contains a heparin‐binding domain sequestered by the low affinity–high avidity sites widespread among the extracellular matrix proteoglycans (Kemp, Mulloy, & Gherardi, 2006; Rubin et al., 2001). These proteoglycan binding sites prevent effective biodistribution of the molecule after injection. Compared with the natural HGF ligand, the agonist mAb does not require activation and circulates freely in the blood and extracellular fluids. The antibody mimics the ligand through its divalent structure, responsible for receptor activation by dimerization.

Anthracyclines are highly effective and widely exploited drugs for cancer treatment. However, their use is limited by the cumulative dose‐dependent cardiotoxicity. Thus, identification of cardioprotective molecules represents an, so far, unmet clinical need (Bell et al., 2016). The MET receptor is dispensable for heart development but is required to maintain cardiac homeostasis, protecting cardiomyocytes from age‐related oxidative stress, apoptosis, and fibrosis (Arechederra et al., 2013). In the present study, we showed that activation of MET signalling by an agonist antibody protected the left ventricle from doxorubicin‐induced contractile dysfunctions, in EF, SV, and CO. A previous study based on HGF gene therapy suggested that MET activation might be beneficial to protect mice from acute doxorubicin‐induced cardiotoxicity (Esaki et al., 2008). We now show that antibody‐mediated activation of MET is exploitable in the prophylaxis of chronic cardiotoxicity. The mouse model of doxorubicin‐induced cardiotoxicity, used in our work, did not show marked histopathological changes, such as myofibrillar degeneration and extensive vacuolization reported in another model of chronic doxorubicin administration (Yi et al., 2006). These animals differ from our own as both the cumulative dose (30 vs. 21 mg·kg^−1^), reached at the end of the treatment, and the number of treatment repetitions (12 compared with 3 once a week) were higher. The animals treated with a single acute dose of doxorubicin (20 mg·kg^−1^; Olson et al., 2003) also show more severe histopathological changes than those treated chronically. Thus, the timing and dose of doxorubicin may give rise to the observed differing severity of histopathological lesions shown in the various animal models. In our model, the chronic cardiac injury was histologically and molecularly characterized by fibrosis, which was pronounced after doxorubicin treatment. Accumulation of extracellular matrix is likely to increase the workload of cardiomyocytes and to cause a decline of cardiac contractility and left ventricular EF. Importantly, we show that treatment of mice with the agonist mAb mitigated the doxorubicin‐induced pro‐fibrotic and remodelling response, confirming the well‐known anti‐fibrotic role of MET.

The signalling pathways involved in MET‐mediated cardioprotection in vivo have not been conclusively established. The long‐term analysis (3 weeks from last Doxo + mAb treatment) did not detect phosphorylation of signalling proteins downstream of the MET receptor (data not shown). Activation of pathways downstream of MET, by mAb were undetectable also in the short‐term analysis (48 hr from doxorubicin treatment and 72 hr from mAb treatment), suggesting that activation was likely to have occurred early, during, and before treatment time. The STAT3 phosphorylation and signalling was increased in both Doxo and Doxo + mAb groups of mice. Several studies suggest that STAT3 is required to protect cells from cardiac stressors. Selective knockout of the *Stat3* gene is followed by cardiac stress adaptation, pathological remodelling, and heart failure (Hilfiker‐Kleiner et al., 2004; Jacoby et al., 2003; Kunisada et al., 2000; Zhang et al., 2016). It is thus conceivable that, through STAT3 stimulation, the heart could trigger an intrinsic compensatory and protective response to doxorubicin treatment. However, prolonged STAT3 activation is detrimental in the setting of myocardial infarction, suggesting that precise and time‐regulated STAT3 activation is required for beneficial effect (Hilfiker‐Kleiner et al., 2004).

Because the in vivo analysis of MET expression suggested that MET activation probably affected cardiomyocytes, we set up an in vitro cardiotoxicity model by treating H9c2 cardiomyoblasts with doxorubicin and two MET agonists (HGF and the mAb). This experimental model allowed us to confirm the cardioprotective effect of MET on cardiomyocytes. In previous work, we demonstrated that activation of the MET receptor protected cardiac cells from hypoxic injury through inhibition of apoptosis (Gallo et al., 2014). Here, we showed that treatment of cardiomyocytes with mAb mitigated the doxorubicin‐induced pro‐apoptotic response. There is much evidence that cardiomyocyte apoptosis is dependent on caspase 3. Our data showed that the agonist mAb protected cardiomyocytes by reducing (a) the expression of PARP1, (b) the phosphorylation of H2AX, and (c) the cleavage of caspase 3. Persistence of γH2AX is considered a surrogate of cell killing by lack of repair of DNA double‐strand breaks (Banáth & Olive, 2003). We previously reported that activation of MET protected against DNA damage induced by ionizing radiation. In glioma cells, irradiation induces ligand‐independent overexpression and activation of MET (De Bacco et al., 2011). In line with these observations, we now found that, in cardiomyocytes, activation of MET protected against DNA damage. Thus, besides defence from hypoxia, the geno‐protective effect of the HGF–MET axis represents a cellular mechanism of adaptation to stressful environmental conditions. Indeed, any signalling pathway exerting a pro‐survival function in normal tissues may be usurped by oncogene products in cancer cells (Hanahan & Weinberg, 2011). The heart is vulnerable to the inhibition of pathways which are targeted in cancer, as in the case of HER2 therapy (Eschenhagen et al., 2011). MET‐targeted therapies, which include HGF‐neutralizing antibodies, MET‐down‐regulating antibodies, and MET tyrosine kinase inhibitors, are currently being exploited as powerful strategies to treat tumours (Comoglio, Trusolino, & Boccaccio, 2018). Considering the critical role played by the HGF–MET axis in myocardial protection, a warning should be given about the possible cardiac side effects of the cancer target therapies.

As well as in the case of protection from hypoxia, the detailed mechanistic explanation of protection from apoptosis and genotoxicity are largely unknown. This work suggests that STAT3 plays a role in MET‐driven cardioprotection in vitro. STAT3 is one of the downstream signalling molecules activated by the HGF–MET axis, and activation of STAT3 has been reported to trigger MET‐dependent branching morphogenesis (Boccaccio et al., 1998), wound healing (Sano, Yoshikawa, Itami, & Takeda, 2001), invasion (Cramer et al., 2005), and anchorage‐independent growth (Kermorgant & Parker, 2008; Zhang, Wang, Jove, & Vande Woude, 2002). Epithelial cells undergoing MET‐driven morphogenesis are protected from “anoikis,” a form of apoptotic cell death occurring as a consequence of loss of adhesion to the extracellular matrix (Frisch & Francis, 1994). Here, we show that STAT3 is part of a mechanism to ensure DNA integrity in cardiomyocytes. However, the in vitro results are different from the in vivo analysis. In fact, in vitro STAT3 phosphorylation was not increased by doxorubicin alone. This discrepancy could be explained by different exposure timing to doxorubicin (1 hr in vitro vs. 48 hr in vivo). Alternatively, different cardiac cell populations may contribute to the doxorubicin response in vivo. In fact, in addition to cardiomyocytes, endothelial cells, smooth muscle cells, fibroblasts, and inflammatory cells may modulate the STAT3 signalling pathway (Haghikia, Ricke‐Hoch, Stapel, Gorst, & Hilfiker‐Kleiner, 2014). STAT3 is a key regulator of intercellular communication between cardiomyocytes, the cardiac micro‐environment, and the extracellular matrix (Haghikia et al., 2014).

The favourable effects of MET activation, resulting from the administration of our agonist mAb, encourage its use in lowering the adverse effects of anthracyclines in cancer therapy. Although some caveats should be considered including possible tumour protecting effect, interventions to alleviate chemotherapy toxicity could be envisaged in patients with a favourable risk to benefit balance. Here, we propose a time‐regulated administration of the MET activating antibody. One dose 24 hr before doxorubicin treatment is sufficient to alert cardiac cells to repair DNA damage, mitigating cardiac injury. However, further investigation is required to address whether this short treatment regimen may affect the anti‐cancer efficacy of doxorubicin. The short‐term MET activation by mAb should be an advantage, as the emergence of MET‐driven tumour clones derives only from long‐term paracrine stimulation (Comoglio et al., 2018). Thus, the humanized MET agonist antibody could be administered as prophylactic, preventive therapy in ambulatory cancer patients who are receiving chemotherapy. However, potential side‐effects, such as malignancies due to secondary resistance, might arise in tumours carrying MET amplification and/or MET overexpression. Precise identification of *MET* genetic lesions will be required to select the oncological patients that would have the benefit clearly outweighing the amount of risk.

## AUTHOR CONTRIBUTIONS

S.G. performed and analysed the in vivo treatments and immunofluorescence experiments. Furthermore, she contributed to (a) the conception and design of the research, (b) collection and interpretation of data, and (c) manuscript writing. M.S. performed western blot experiments and mRNA quantitative analysis. R.A. produced and purified the MET agonist monoclonal antibody and performed immunohistochemical staining. G.F. and E.D.G. performed and analysed MRI. E.C. performed and provided flow cytometry data. D.B. performed histopathological analysis. A.B. contributed to the analysis and interpretation of cardiac functional parameters. C.B. and A.S. assisted with the interpretation of data. P.M.C. and T.C. contributed to (a) the conception and design of the research, (b) interpretation of data, and (c) manuscript writing.

## CONFLICT OF INTEREST

C.B. and P.M.C. own shares in Metis BeCorp. The other authors declare no conflict of interest.

## DECLARATION OF TRANSPARENCY AND SCIENTIFIC RIGOUR

This Declaration acknowledges that this paper adheres to the principles for transparent reporting and scientific rigour of preclinical research as stated in the *BJP* guidelines for Design & Analysis, Immunoblotting and Immunochemistry, and Animal Experimentation, and as recommended by funding agencies, publishers, and other organizations engaged with supporting research.

## Supporting information

Table S1. Evaluation of mice organ weight

Table S2. TaqMan gene expression assay probes used in the study

Table S3. Antibodies used throughout the study

Figure S1 Supporting Information

Figure S2 Supporting Information

Figure S3 Supporting Information

Figure S4 Supporting Information

Figure S5 Supporting Information

Figure S6 Supporting Information

Data S1 Supporting Information
