# Supplementary material for: Activation of the MET receptor attenuates doxorubicin‐induced cardiotoxicity in vivo and in vitro
Source: Br J Pharmacol. 2020 May 29;177(13):3107–22. doi: 10.1111/bph.15039 (PMC7280013; doi:10.1111/bph.15039)
Supplement: Supplementary file 1 — Table S1. Evaluation of mice organ weight [file BPH-177-3107-s001.docx]

**Supplementary Table 1.** Evaluation of mice organ weight.

|  | **Placebo** | **Doxo** | **Doxo+MetAmab** |  |
| --- | --- | --- | --- | --- |
| **Heart Weight (mg)** | 103.04 ± 17.9 | 104.47 ± 25.3 | 111.98 ± 13.05 | |
| **Spleen Weight (mg)** | 76.17 ± 11.5 | 57.63 ± 12.9 ^a^ | 53.69 ± 8.60 ^a^ | |
| **Right Kidney Weight (mg)** | 129.88 ± 21.1 | 98.50 ± 12.3 ^a^ | 100.73 ± 8.36 ^a^ | |
| **Left Kidney Weight (mg)** | 126.79 ± 12.8 | 104.38 ± 12.7 ^a^ | 100.43 ± 12.64 ^a^ | |
| **Liver Weight (g)** | 0.947 ± 0.111 | 0.734 ± 0.186 ^a^ | 0.762 ± 0.087 ^a^ | |
| **Lung Weight (mg)** | 141.09 ± 17.6 | 139.44 ± 15.28 | 133.26 ± 20.32 | |

All the values are normalized on relative Tibial Lenghts.

^a^ Significant vs Control
