# Supplementary material for: Activation of the MET receptor attenuates doxorubicin‐induced cardiotoxicity in vivo and in vitro
Source: Br J Pharmacol. 2020 May 29;177(13):3107–22. doi: 10.1111/bph.15039 (PMC7280013; doi:10.1111/bph.15039)
Supplement: Supplementary file 2 — Table S2. TaqMan gene expression assay probes used in the study [file BPH-177-3107-s002.docx]

**Supplementary Table 2.** TaqMan gene expression assay probes used in the study.

| **Gene** | **TaqMan Probes** |
| --- | --- |
| Ctgf | Mm01192933_g1 |
| Col1a2 | Mm00483888_m1 |
| Acta1 | Mm00808218_g1 |
| NPPA | Mm01255747_g1 |
| Timp1 | Mm00441818_m1 |
| Mmp9 | Mm00442991_m1 |
| IL-6 | Mm00446190_m1 |
| Met | Mm01156972_m1 |
| HGF | Mm01135184_m1 |
| Polr2a | Mm00839493_m1 |
