# Supplementary material for: Activation of the MET receptor attenuates doxorubicin‐induced cardiotoxicity in vivo and in vitro
Source: Br J Pharmacol. 2020 May 29;177(13):3107–22. doi: 10.1111/bph.15039 (PMC7280013; doi:10.1111/bph.15039)
Supplement: Supplementary file 3 — Table S3. Antibodies used throughout the study [file BPH-177-3107-s003.docx]

**Supplementary Table 3.** Antibodies used throughout the study.

| **Primary Antibody** | **Company** | **Dilution** | **Species** | **Isotype** | **Product n.** | **Exp** | **RRID** |
| --- | --- | --- | --- | --- | --- | --- | --- |
| α-SMA | Cell Signaling | 1:1000 | Rabbit | IgG | 19245 | WB | RRID:AB_2734735 |
| P-Erk1,2 (Thr-202/Tyr-204) | Cell Signaling | 1:1000 | Rabbit | IgG | 4376 | WB | RRID:AB_331772 |
| Erk2 | Santa Cruz | 1:1000 | Rabbit | IgG | sc-154 | WB | RRID:AB_2141292 |
| P-Akt (Ser-473) | Cell Signaling | 1:1000 | Rabbit | IgG | 9271 | WB | RRID:AB_329825 |
| Akt | Cell Signaling | 1:1000 | Rabbit | IgG | 9272 | WB | RRID:AB_329827 |
| P-Stat3 (Tyr-705) | Cell Signaling | 1:1000 | Rabbit | IgG | 9145 | WB | RRID:AB_2491009 |
| Stat3 | Cell Signaling | 1:1000 | Mouse | IgG2a | 9139 | WB | RRID:AB_331757 |
| γH2AX (Ser-139) | Cell Signaling | 1:1000/1:200 | Rabbit | IgG | 9718 | WB | RRID:AB_2118009 |
| H2AX | Cell Signaling | 1:1000 | Rabbit | IgG | 2595 | WB | RRID:AB_10694556 |
| Parp1 | Cell Signaling | 1:1000 | Rabbit | IgG | 9541 | WB | RRID:AB_331426 |
| Total/Cleaved Caspase 3 | Cell Signaling | 1:1000 | Rabbit | IgG | 9662 | WB | RRID:AB_331439 |
| Cleaved Caspase 3 | Cell Signaling | 1:200 | Rabbit | IgG | 9579 | IF | RRID:AB_10897512 |
| Met | R&D | 1:200 | Goat | IgG | AF527 | IHC | RRID:AB_355414 |
| Stat3 | Cell Signaling | 1:50 | Rabbit | IgG1 | 30835 | IHC | RRID:AB_2798995 |
| αTubulin | Sigma | 1:1000 | Mouse | IgG1 | T5168 | WB | RRID:AB_477579 |
|  |  |  |  |  |  |  |  |
| **Secondary Antibody** |  |  |  |  |  |  |  |
|  |  |  |  |  |  |  |  |
| Goat anti-mouse IG | Jackson | 1:5000 | Goat | IgG | 115-035-003 | WB | RRID:AB_10015289 |
| Goat anti-rabbit IG | Jackson | 1:5000 | Goat | IgG | 111-035-003 | WB | RRID:AB_2313567 |
| Alexa Fluor 555 Phalloidin | Invitrogen | 1:400 | na | na | A34055 | IF | na |
| Alexa Fluor 488 anti-rabbit | Invitrogen | 1:400 | Donkey | IgG | A21206 | IF | RRID:AB_141708 |
