# Supplementary figures and images for: Activation of the MET receptor attenuates doxorubicin‐induced cardiotoxicity in vivo and in vitro
Source: Br J Pharmacol. 2020 May 29;177(13):3107–22. doi: 10.1111/bph.15039 (PMC7280013; doi:10.1111/bph.15039)

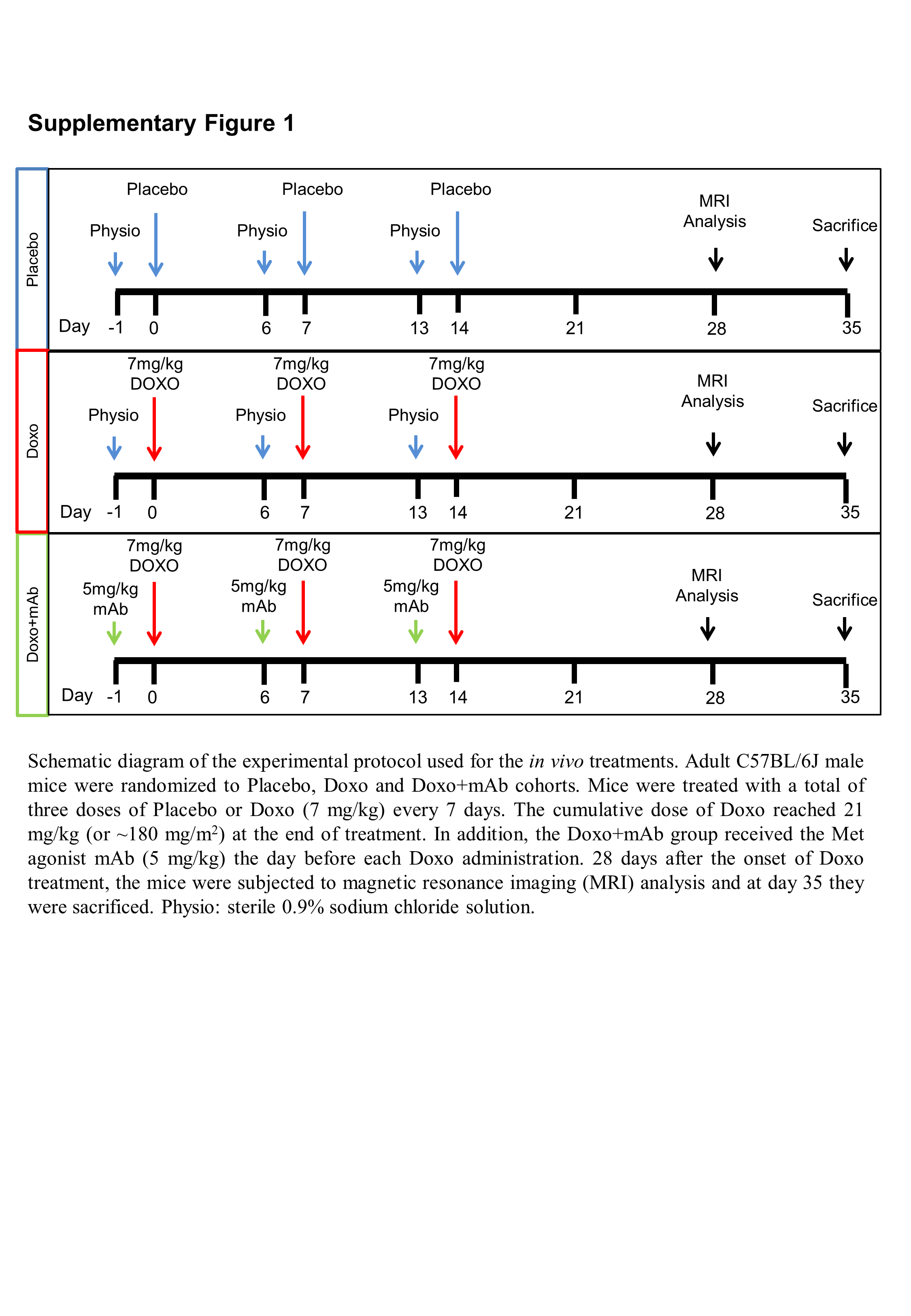

Supplement: Supplementary file 4 — Figure S1 Supporting Information [file BPH-177-3107-s004.tif]

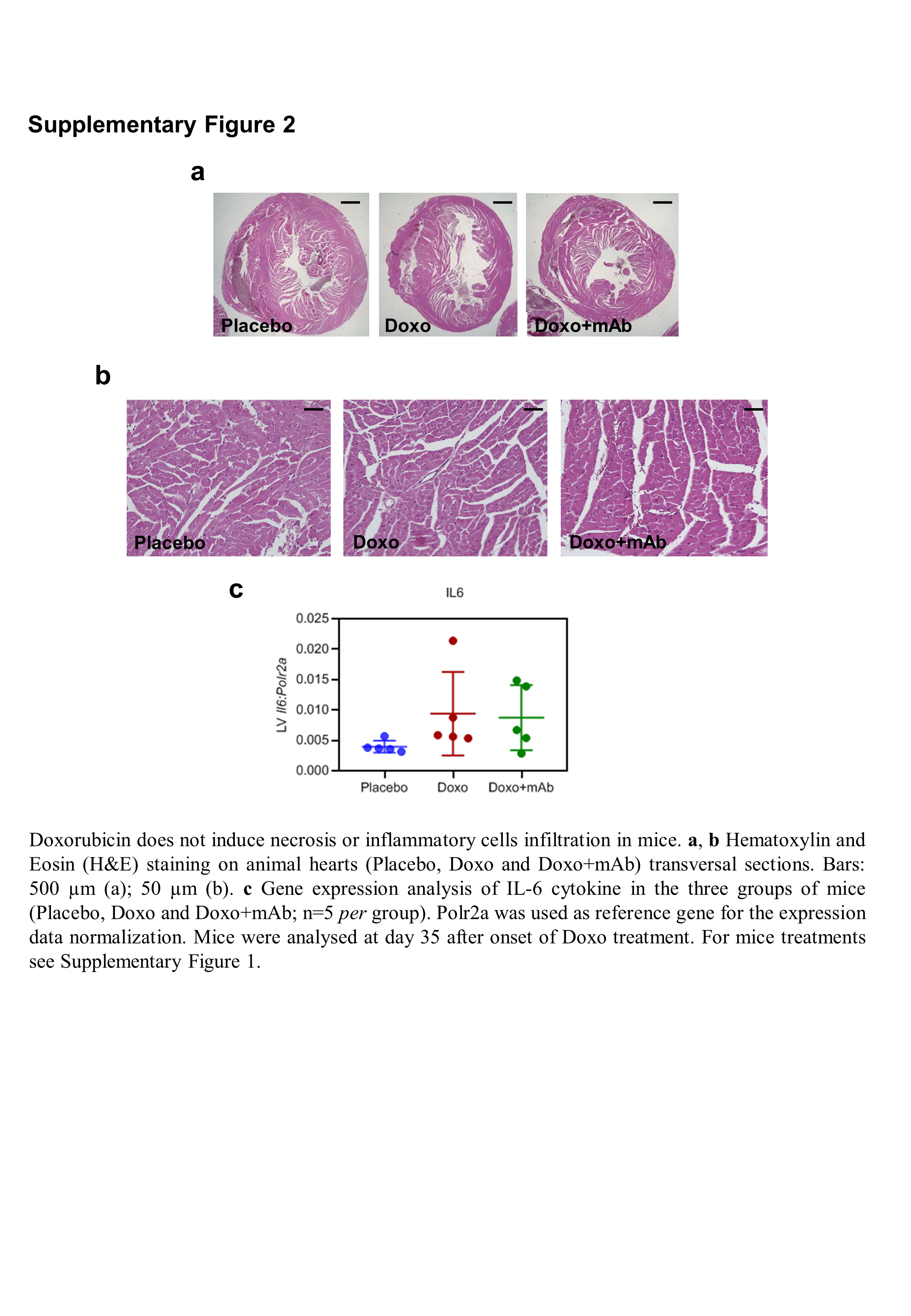

Supplement: Supplementary file 5 — Figure S2 Supporting Information [file BPH-177-3107-s005.tif]

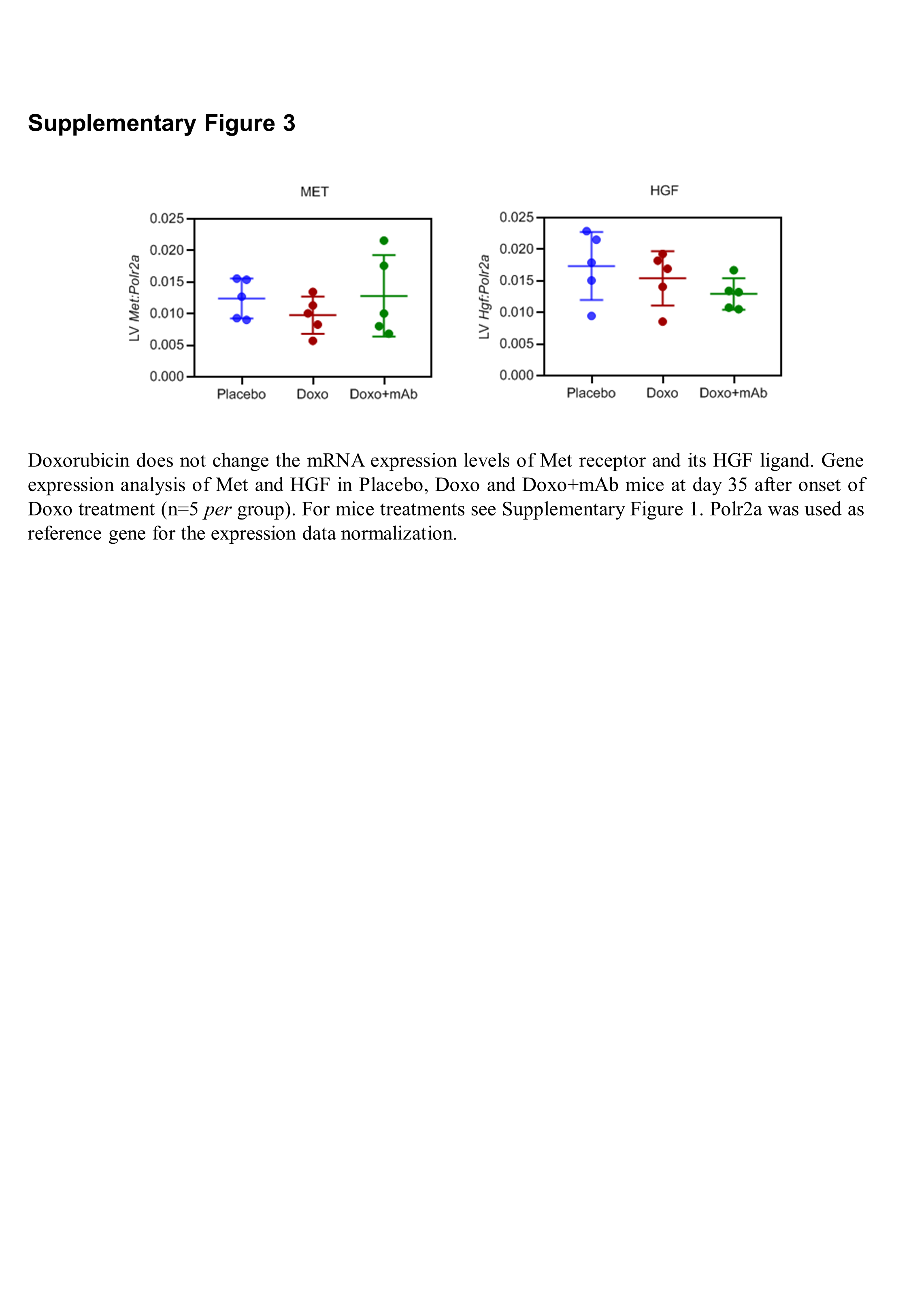

Supplement: Supplementary file 6 — Figure S3 Supporting Information [file BPH-177-3107-s006.tif]

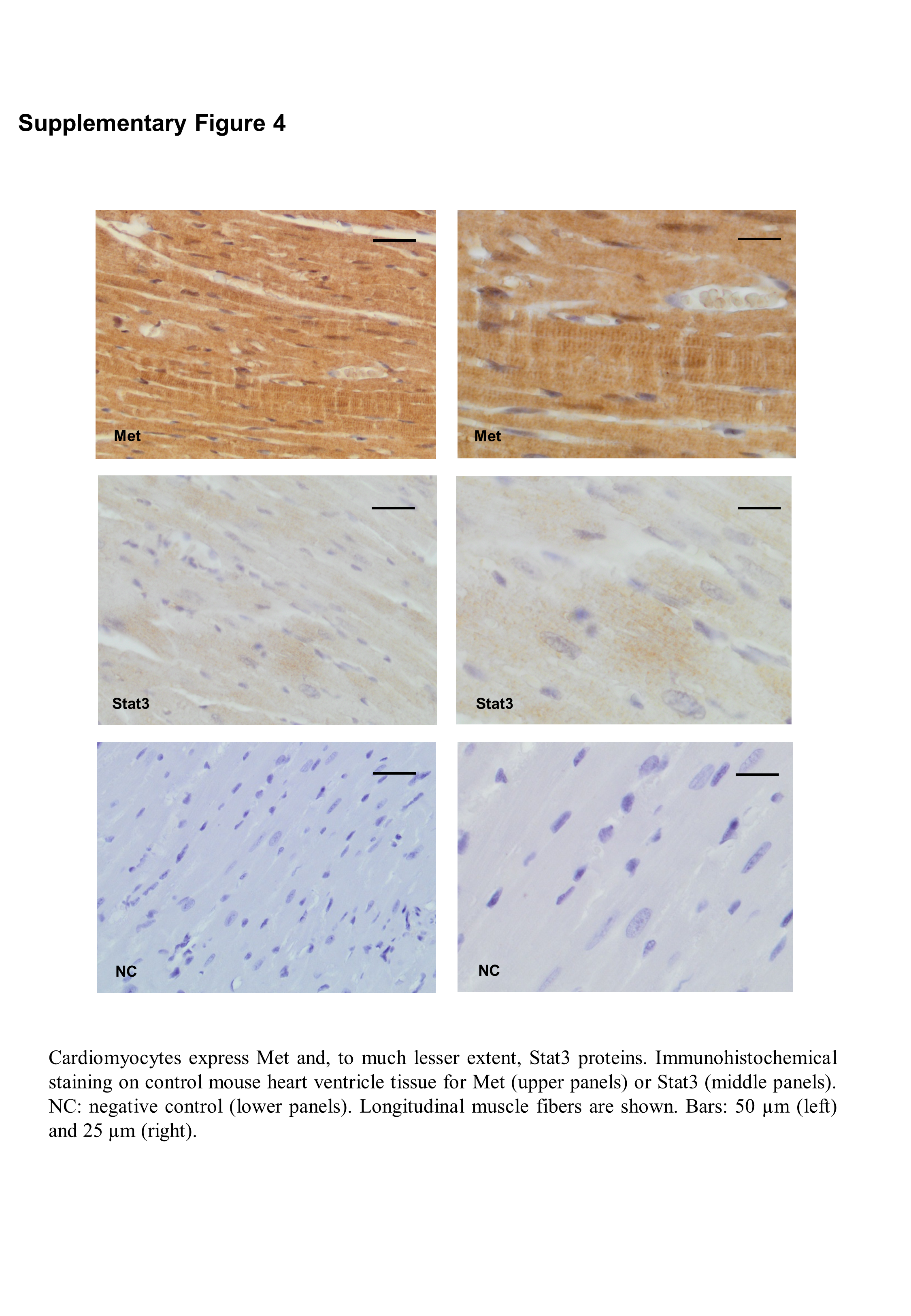

Supplement: Supplementary file 7 — Figure S4 Supporting Information [file BPH-177-3107-s007.tif]

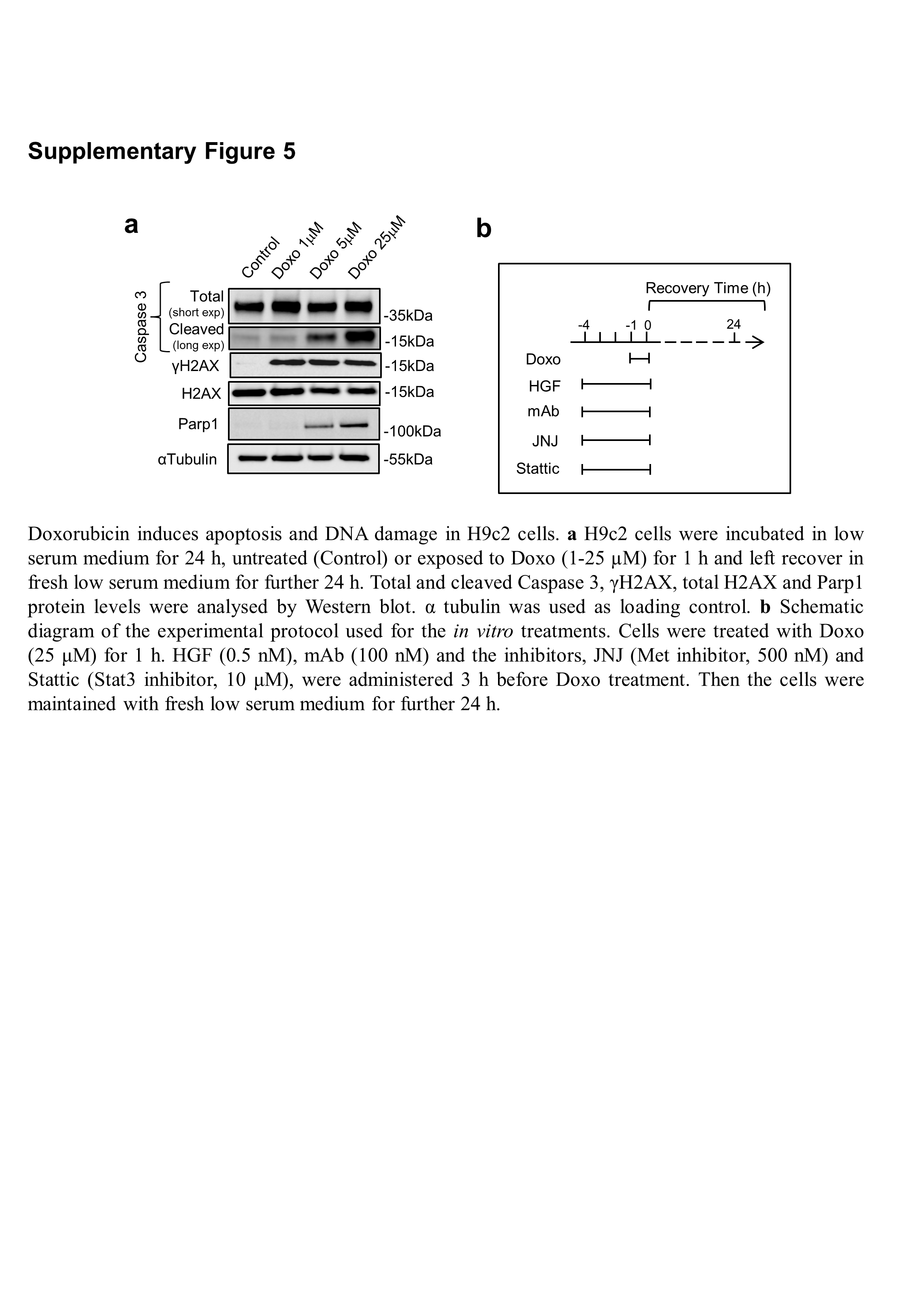

Supplement: Supplementary file 8 — Figure S5 Supporting Information [file BPH-177-3107-s008.tif]

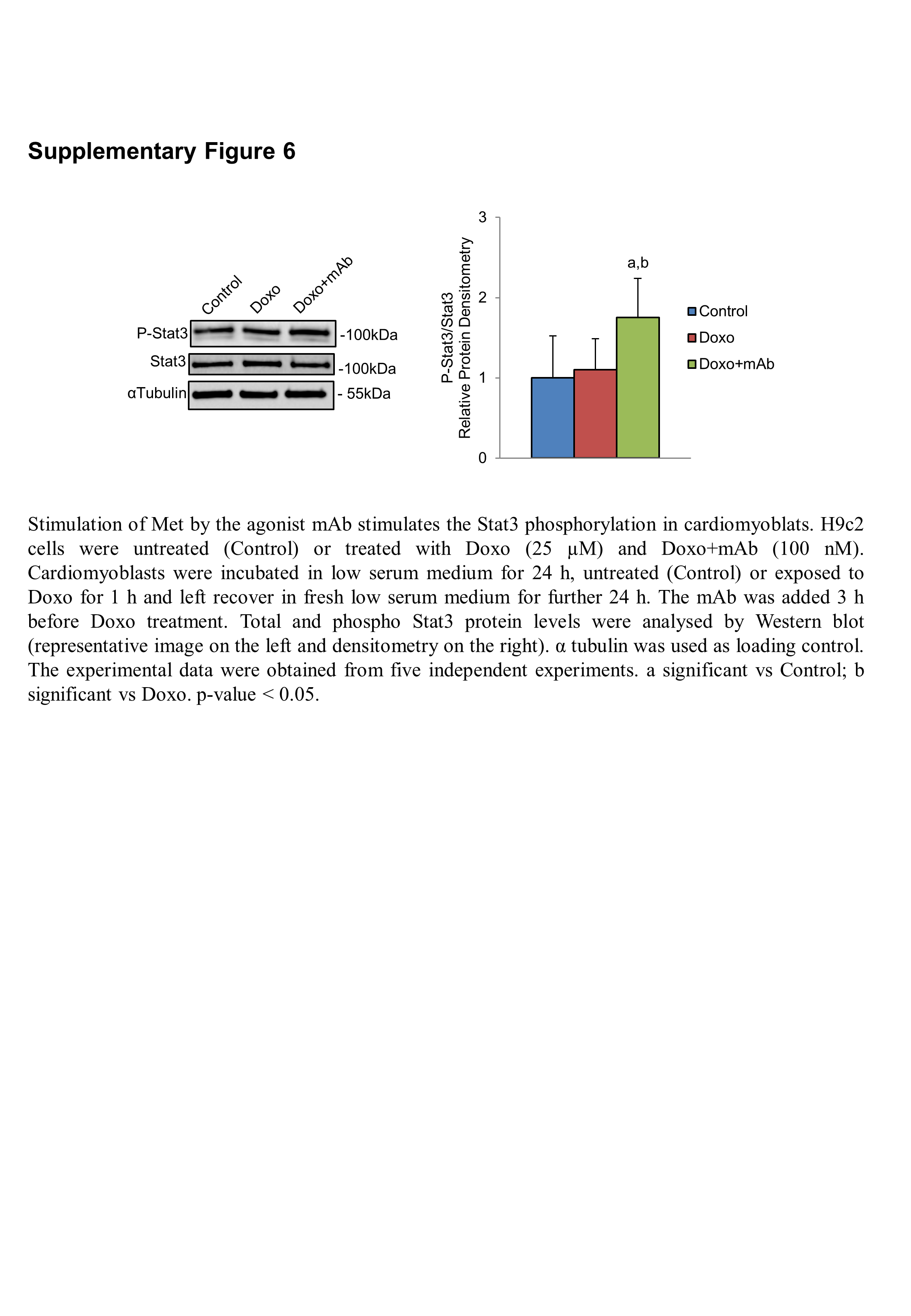

Supplement: Supplementary file 9 — Figure S6 Supporting Information [file BPH-177-3107-s009.tif]
