# Supplementary material for: Activation of the MET receptor attenuates doxorubicin‐induced cardiotoxicity in vivo and in vitro
Source: Br J Pharmacol. 2020 May 29;177(13):3107–22. doi: 10.1111/bph.15039 (PMC7280013; doi:10.1111/bph.15039)
Supplement: Supplementary file 10 — Data S1 Supporting Information [file BPH-177-3107-s010.pdf]

Supplementary Methods

Immunohistochemistry Analysis

PBS-rinsed hearts were fixed in freshly made Formalin (Sigma-Aldrich) overnight at 4°C and then processed with 4 cuts parallel and transversal to the tip, the midline of the ventricles, the base and the atria. After processing the tissue were embedded in paraffin (Leica Microsystems, Wetzlar, Germany). 5 μm thick serial sections, were deparaffinized, rehydrated and stained with Hematoxylin & Eosin (Bio-Optica, Milan, Italy) for morphological evaluation (Supplementary Figure 3). For Stat3 and Met staining (Supplementary Figure 4) in heart horizontal sections at mid-level antigens were unmasked in Citrate Buffer at pH 6.00, heating at 800W in microwave for 5 minutes and for 20 additional minutes in a water bath at 95°C (Stat3 staining) or for 1 hour (Met staining) in a water bath at 95°C. After heating the slides were cooled for 30 minutes. Hydrogen peroxide 3% in TBS was employed for 10 minutes to quench endogenous peroxidases. Tissue sections were incubated for 1 hour at RT in a blocking solution (5% BSA in TBS-Tween-Triton) and overnight at 4°C with the appropriate primary antibody (see Supplementary Table 3). The following day, tissue sections were incubated for 1 hour at RT with Anti-Goat-HRP (Agilent Dako, Santa Clara, CA, USA) for Met staining and with Anti-Rabbit-HRP (Agilent Dako) for Stat3 staining. Peroxidase activity was developed with DAB (ImmPACT DAB, Vector, Burlingame, CA, USA). The chromagenic reaction was stopped by milliQ H_2O. Nuclear counterstaining was performed using Hematoxylin (Bio-Optica, Milan, Italy). Stained tissue sections were dehydrated and mounting media was added to apply the coverslips. Images were taken with Leica ICC50 microscope at 2.5X, 20X and 40X magnification. LAS AF Leica software was used for acquisition. The experimental data on immunohistochemistry analysis conform with BJP Guidelines (Alexander *et al*., 2018).
